# Genetic Risk Profiling Associated with Recurrent Unprovoked Venous Thromboembolism

**DOI:** 10.3390/genes12060874

**Published:** 2021-06-07

**Authors:** Hossam Hodeib, Amira Youssef, Alzahraa A. Allam, Amal Selim, Mohamed A. Tawfik, Mohammed F. Abosamak, Ahmed Esam, Mohamed S. Abd Elghafar, Sameh Samir, Ola A. ELshora

**Affiliations:** 1 Clinical Pathology Department, Faculty of Medicine, Tanta University, Tanta 31512, Egypt; hossamhodeib@gmail.com (H.H.); Damirayoussef@yahoo.com (A.Y.); ola.a.elshora@gmail.com (O.A.E.); 2 Internal Medicine Department, Faculty of Medicine, Tanta University, Tanta 31512, Egypt; alzahraa.allam@gmail.com (A.A.A.); amalibrahims@hotmail.com (A.S.); 3 Anesthesia/ICU Department, Faculty of Medicine, Tanta University Hospital, Tanta 31512, Egypt; Samakawy10@yahoo.com (M.F.A.); madamada6@gmail.com (A.E.); mohamed.abdelghafar1@med.tanta.edu.eg (M.S.A.E.); 4 Cardiovascular Medicine Department, Faculty of Medicine, Tanta University, Tanta 31511, Egypt; drsameh76@gmail.com

**Keywords:** venous thromboembolism, genetic risk score

## Abstract

Introduction: Venous thromboembolism (VTE), including deep vein thrombosis (DVT) and/or pulmonary embolism (PE), is a common, acute, multifactorial disease with a five-years cumulative incidence of recurrence of approximately 25%. Actually, no single genetic defect can predict the risk of recurrence of VTE. Therefore, individual genetic risk profiling could be useful for the prediction of VTE recurrence. Aim of the study: To assess the combined effect of the common prothrombotic genotypes on the risk of recurrence of VTE in recently diagnosed unprovoked VTE patients. Patients and methods: This population based, prospective follow-up study was carried out from January 2015 to December 2020 in (internal medicine, cardiovascular medicine and anesthesia and ICU departments, Tanta University Hospital, Egypt) on 224 recently diagnosed unprovoked VTE patients. Whole blood was collected by standard venipuncture at the time of admission prior to the beginning of anticoagulant therapy. Genomic DNA was extracted and was genotyped for the 5-SNPs Genetic risk score (GRS), previously validated for first venous thrombosis (FVL rs6025, PTM rs1799963, ABO rs8176719, FGG rs2066865 and FXI rs2036914). Results: The main important finding in the present study was that patients having ≥3 risk alleles were associated with higher risk of VTE recurrence compared to those having ≤2 risk alleles (the reference group) (HR 2.5, 95% CI 1.48–4.21) (*p* = 0.001). Patients with GRS ≥ 3 had a significantly shorter time recurrence free survival (43.07 months) compared to the low risk group of patients with GRS (0–2) (*p* < 0.001). Conclusion: GRS model could be an effective and useful model in risk stratification of VTE patients, and genetic risk profiling of VTE patients could be used for the prediction of recurrence of VTE.

## 1. Introduction

Venous thromboembolism (VTE), including deep vein thrombosis (DVT) and/or pulmonary embolism (PE), is a common, multifactorial disease, with a five-years cumulative incidence of recurrence of approximately 25% [1,2]. Thrombophilia is a typical multifactorial disease; the pathogenesis of VTE involves genetic and environmental risk factors [3]. In familial thrombophilia, gene–gene and gene–environment interactions play a vital role; coexistence of different prothrombotic defects increases the risk of developing VTE either in the presence or the absence of transient acquired risk factors. This means that inherited thrombophilia, differently from inherited hemophilia, does not follow the single-gene defect model. In brief, the multifactorial and polygenic nature of VTE, in which inherited and acquired conditions act in synergism, strongly modulate the onset and progression of VTE [4,5]. The most prominent genetic risk factors for VTE are deficiencies of the natural anticoagulants (antithrombin, protein C and protein S), Factor V Leiden (FVL), Factor II G20210A mutation (PTM), ABO, fibrinogen-γ (FGG) and Factor XI (FXI) mutations. Acquired risk factors associated with VTE comprise major surgery, malignancy, hormonal replacement therapy, antiphospholipid syndrome, myeloproliferative disorders, age, trauma and immobilization [6].

Anticoagulant therapy is an effective treatment for the first thrombotic event as well as for prevention of recurrence of VTE with a minimum duration of 3 months [7]. Continuation of anticoagulant therapy after the initial treatment of VTE decreases the risk of recurrence; however, it should be carefully done to maintain the hemostatic balance and guard against the catastrophic side effects, i.e., anticoagulant therapy-related bleeding [8,9,10]. The current guidelines advise to stratify VTE patients into only two groups, with either high (unprovoked) or low (provoked) risk of VTE recurrence. Simply, the provoked/low-risk group is advised to stop the anticoagulant therapy and the unprovoked/high-risk group is advised to continue unless bleeding risk is expected [11,12]. VTE is a complex multifactorial disease with integrating genetic and environmental risk factors. The well-known genetic risk factors, Factor V Leiden (FVL), Factor II G20210A mutation (PTM), protein C, protein S and antithrombin deficiency, does not appear to predict the risk of recurrence of VTE [13,14,15]. Indeed, the genetic defects involved in the thrombotic disorders with their complexity of underlying mechanisms represents a major great obstacle to ascribe to the classical causative-gene-defect-model that can predict the risk of recurrence of VTE. Therefore, individual genetic risk profiling could be useful for the prediction of VTE recurrence [16,17]. Van Hylckama Vlieg et al. proposed a simplified genetic risk score model (5-SNP GRS), i.e., FVL rs6025, PTM rs1799963, ABO rs8176719, fibrinogen-γ (FGG rs2066865) and FXI rs2036914, which could be useful for risk classification of VTE patients [18]. Unfortunately, a little information in the literature discusses this point exactly, and most studies were performed at a single center with patients of similar ethnic background and applied to patients of white descent.

The aim of this study was to assess the combined effect of the common prothrombotic genotypes (the five genetic variants that were most strongly associated with the risk of a first venous thrombosis in literature) on the risk of recurrence of VTE in recently diagnosed unprovoked VTE patients.

## 2. Patients and Methods

This population based, prospective follow-up study was carried out from January 2015 to December 2020 in (internal medicine, cardiovascular medicine and anesthesia and ICU departments, Tanta University Hospital, Egypt) on 224 patients of both sexes, aged ≥18 years at the time of first thrombotic event. The study followed Guidelines regarding participation, drug administration and sample collection which meet the requirements of the declaration of Helsinki. The ethical committee of Tanta University Hospital approved the study protocol and the written consent form by volunteers.

They were diagnosed as unprovoked VTE patients. DVT was confirmed by objective methods: venography or compression ultrasonography. PE was confirmed by a ventilation/perfusion lung scan, spiral computed tomography or angiogram. All details about the patients were recorded within the patients’ files: date of occurrence of the first thrombotic event, site of the episode and presence of other situations predisposing to DVT or PE in the months preceding the event. Our patients were recently diagnosed as unprovoked VTE patients. They were defined as none of them had any of the provoking factors: surgery, trauma, hospitalization, plaster cast, immobilization (bed confinement for at least 7 days), hormonal replacement therapy, and pregnancy within 3 months preceding the first event, puerperium (4 weeks after delivery) or malignancy within 5 years before the first event. VTE patients were treated according to the standard protocol; they were initially treated with LMWH then with warfarin as OAC for at least 3 months. All patients were eligible for the study after approval by the hospital ethical committee, and the study was performed according to the principles of the declaration of Helsinki; a written informed consent of the patients was obtained. All patients were invited to enter the study at the time of first thrombotic event; they were observed for a period of five years at three-month intervals in the first year and every six months in the following years until the date of recurrence, death of the patient due to recurrent fatal PE (counted as recurrent event) or the end of the five years which is the end point of the study.

### 2.1. Blood Collection, DNA Extraction and Genotyping

Whole blood was obtained at the time of admission prior to the beginning of anticoagulant therapy. Whole blood was collected by standard venipuncture, and genomic DNA was extracted by using QIAamp DNA Mini Kit (Cat No: 51306, QIAGEN GmbH, Hilden, Germany) DNA samples were stored at −20 °C until the time of the assay. DNA samples were genotyped for FVL rs6025, PTM rs1799963, ABO rs8176719, FGG rs2066865 and FXI rs2036914 on Applied Biosystems StepOne™Real-Time PCR Systems (Applied Biosystems, Foster City, CA, USA) at molecular biology unit, clinical pathology department, Tanta university hospital, Egypt. In brief, Predesigned/Custom TaqMan^®^ SNP Genotyping Assays kit (catalog No.4351379/4331349, Thermo Fisher Scientific, Waltham, MA, USA) for FVL rs6025, PTM rs1799963, ABO rs8176719, FGG rs2066865, and FXI rs2036914 were designed to identify the point substitutions in the respective genes. PCR reaction (Polymerase Chain Reaction) was done in a final volume of 25 µL 5 µL of purified DNA was added to a volume of 20 µL of the amplification mix according to manufacturer’s instructions. Thermal profile for PCR was programmed as follow: denaturation at 95 °C for 10 min (Polymerase activation), followed by 40 cycles of 95.5 °C for 15 s (denaturation) and 60°C for 1 min (Annealing/extension). Data was analyzed and SNPs were determined.

### 2.2. Statistical Analysis

Data were fed to the computer and analyzed using IBM SPSS software package version 20.0. (Armonk, NY, USA: IBM Corp). The Kolmogorov–Smirnov was used to verify the normality of distribution of variables. Comparisons between groups for categorical variables were assessed using Chi-square test (Monte Carlo). Student *t*-test was used to compare two groups for normally distributed quantitative variables. Mann–Whitney test was used to compare two groups for abnormally distributed quantitative variables. For normally distributed variables, data was presented as mean ± SD whereas for asymmetrical variable, data was presented as median and IQR (interquartile range). For the individual associations of each of the five SNPs with the risk of recurrent VTE, Cox regression models were performed to calculate the hazard ratios (HR) with 95% confidence intervals (95% CI) either univariate and multivariate (adjusted for age and sex). We calculated the number of risk-increasing alleles carried and compared this between patients with and without a recurrence. In addition, the risk of recurrent VTE associated with the 5-SNP (GRS) was calculated after stratification of the whole group of patients according to the number of risk increasing alleles associated with recurrent VTE (GRS) into high and low risk group by calculating HRs, adjusted for age and sex. Kaplan–Meier curve was obtained to estimate the recurrence-free survival of the five SNPs (GRS) in the whole group of patients. Significance of the obtained results was judged at the 5% level.

## 3. Results

**Figure 1:**
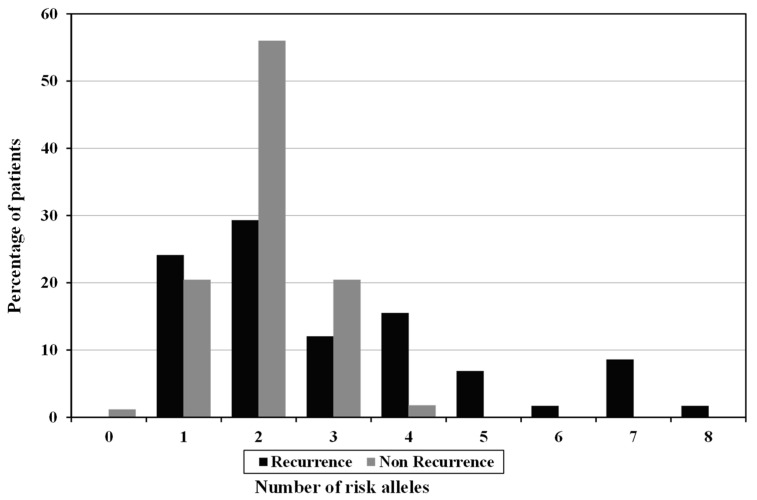
Risk Alleles distribution among recurrent and non-recurrent VTE patients.

In total, 224 patients recently diagnosed as unprovoked VTE, 51.8% were females, 48.2% were males, and the mean of age (At the time of the diagnosis of the 1st thrombotic event) was 51.7 years ± 6.0. There were no statistically significant differences between the two studied groups as regard the gender and age (*p* = 0.991 and *p* = 0.650, respectively) (Table 1). They were followed for 49.57 ± 19.39 months. VTE recurred in 58 (25.8%) patients and 166 (74.2%) were recurrence-free during the follow-up period. VTE patients were subjected to three or six months of warfarin as OAC; they were asked to start warfarin on the day one of the predesigned scheduled five days of heparin according to the clinical setting of each patient. There was no detectable difference between the studied groups of patients in respect to the duration of warfarin (*p* > 0.05) suggesting that three months of anticoagulant therapy is sufficient and has the same benefit as six months. The first important finding obtained from the present study was that, by genotyping of the five SNPs; ABO rs8176719, FVL rs6025, PTM rs1799963, FGG rs2066865 and FXI rs2036914, among the two studied groups of patients, FVL mutation was significantly higher in patients with recurrent VTE (*p* = 0.014) (Table 2). The prevalence of risk alleles for each of the five SNPs were studied in Table 3. The risk allele for ABO, FVL, FGG and FXI was significantly frequent in patients with recurrent VTE (*p* = 0.01, *p* = 0.013, *p* = 0.025 and *p* = 0.043, respectively). Theoretically, such patient might have 0 to 2 risk alleles for each SNP. As a consequence, after calculating the overall risk alleles for the five SNPs, such patient might have 0 to10 risk alleles. Results obtained from the present work revealed that the number of risk increasing alleles ranged from 0 to 8 with median value 2. Furthermore, we stratified the whole group of patients according to the sum of the number of risk increasing alleles associated with recurrent VTE (GRS) into high and low risk group. Interestingly, by comparing the two studied groups of patients, patients carrying ≥3 risk alleles were more frequent in the recurrent group of patients compared to those carrying ≤2 risk alleles (*p* < 0.001) (Table 4). An additional interesting finding was that in the five SNPs scores, there was a tendency toward more risk alleles in patients with recurrence compared to patients without recurrence suggesting that the more risk alleles, the higher the risk of recurrence of VTE (Figure 1).

**Figure 2:**
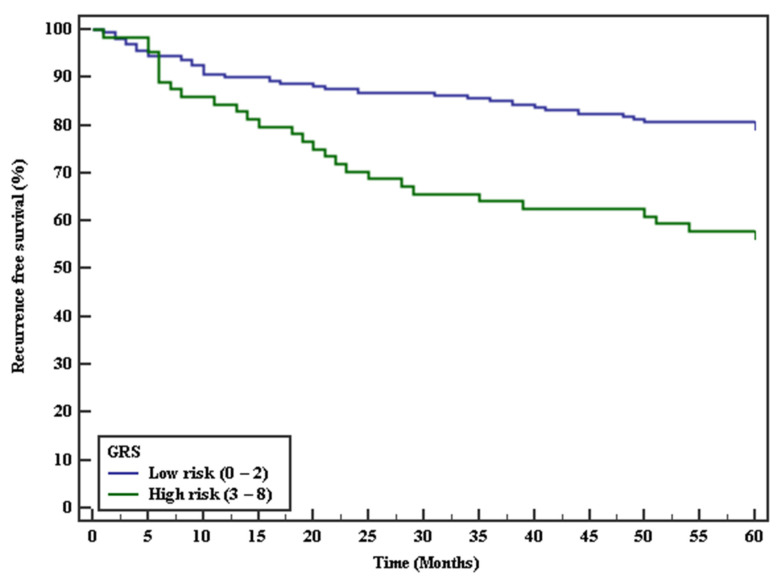
Kaplan–Meier survival curve for recurrence free survival with GRS.

The risk of recurrent VTE associated with the five SNPs was assessed as shown in Table 5. It is noteworthy that the risk of recurrent VTE was significantly higher with FVL mutation (HR 1.95, 95% CI 1.14–3.33) (*p* = 0.015), suggesting that FVL mutation could be an independent risk factor for recurrent VTE. Next, we investigated the potential synergistic effect of different prothrombotic genotypes. We observed that the risk of recurrent VTE associated with combined genetic defects (FVL + PTM), (FVL + FGG), (FVL + FXI) and (FXI + FGG) mutations were (HR 6.83, 95% CI 2.44–19.09), (HR 2.92, 95% CI 1.65–5.15), (HR 5.36, 95% CI 3.05–9.41) and (HR 5.21, 95% CI 3.04–8.94) (*p* < 0.001, respectively). In addition, patients carrying (FVL + ABO), (PTM + ABO), (PTM + FGG), (PTM + FXI), (ABO + FXI) and (ABO + FGG) mutations did not have any significant risk of recurrence (*p* > 0.05, respectively); data are not shown. In addition, patients having ≥3 risk alleles were associated with higher risk of VTE recurrence compared to those having ≤2 risk alleles (the reference group) (HR 2.5, 95% CI 1.48–4.21) (*p* = 0.001) (Table 5) suggesting that our GRS model could be an effective and useful model in risk stratification of VTE patients. Ultimately, Kaplan–Meier curve was plotted to estimate the recurrence-free survival of the five SNPs. Patients with GRS ≥3 had a significantly shorter time recurrence free survival (43.07 months) compared to the low-risk group of patients with GRS (0–2) (*p* < 0.001) as shown in (Table 6, Figure 2). This result confirmed that genetic risk profiling of VTE patients could be used for the prediction of recurrence of VTE.

## 4. Discussion

Risk stratification of VTE patients and the prediction of recurrent VTE would allow proper use of VTE prophylaxis and selective management of high-risk patients. The risk for recurrent VTE varies according to the clinical setting of each patient whether the first event was associated with an acquired risk factor (provoked) or in the absence of any provoking risk factors (unprovoked) [19]. Anticoagulant therapy is the mainstay for the treatment of VTE and very effective at prevention of recurrence, but it is associated with an increased bleeding risk [20]. Once the anticoagulant therapy is started, the risk of VTE recurrence falls rapidly and then falls slowly until a new baseline risk is reached [7,21]. In general, patients with VTE should be treated either for three or six months or indefinitely [22]. Results obtained from the present work revealed no detectable difference between the studied groups in respect to the duration of warfarin (*p* > 0.05). In agreement with our findings, Campbell IA et al. [23] and Agnelli G et al. [24] reported a similar risk of recurrence of VTE with three months compared with six to twelve months of anticoagulant therapy during one to three years of follow-up. On the other side, Levine MN et al. [25], Schulman S et al. [26] and Kearon C et al. [27] demonstrated that the shorter course of anticoagulant therapy was associated with increased risk of recurrence during nine to twenty-four months of follow-up. Genetic risk profiling could be useful in the identification of individuals at high risk for the first thrombotic event as well as the recurrence of VTE. In the present study, we used only the five prothrombotic genotypes that were most strongly associated with the risk of a first venous thrombosis in literature and applied previously to the prediction of first thrombotic events [28] and we developed GRS based on these five SNPs (FVL rs6025, PTM rs1799963, ABO rs8176719, FGG rs2066865 and FXI rs2036914). Both FV and FII have essential role in the coagulation pathway [29] Non-O blood group has an elevated risk of VTE events. It has been attributed to higher levels of FVIII and von Willebrand factor [30,31]. FXI plays an essential role in hemostasis independent of the contact pathway, and it has been described as a strong risk factor in venous thrombosis. Patients with severe FXI deficiency had a lower incidence of VTE. On the contrary, higher levels of FXI were associated with increased VTE risk [32,33]. Low levels of fibrinogen γ (fibrinogen-γ)—a subunit of fibrinogen—have been described as a risk factor for venous thrombosis [34]. Results obtained from the present work revealed that VTE recurred in 58 (25.8%) patients and 166 (74.2%) were recurrence-free during the follow-up period, in addition, neither the gender nor the age had significant differences among the two studied groups of patients (*p* = 0.991 and *p* = 0.650 respectively). This result differs somewhat from that reported by van Hylckama Vlieg et al. and Ahmad A et al. [18,35] who reported the risk of recurrence was higher in men than in women. This difference may be explained by the little number of patients enrolled in the study, or it might be the real picture of the patients in our hospital in that period of time although the underlying mechanism of these findings is still unknown. Interestingly, we found that FVL mutation could be an independent risk factor for recurrent VTE while PTM, ABO, FGG and FXI variants were not. This finding may agree with the fact that FVL mutation is the most prevalent genetic defect associated with venous thrombosis in the literature. Near to our finding, van Hylckama Vlieg et al. and Coppens M [15,18] recorded that neither FVL nor PTM variants were unable separately to predict the risk of VTE recurrence. Additionally, we reported elevated risk of recurrent VTE in patients with double carriers of (FVL + PTM), (FVL + FGG), (FVL + FXI) and (FXI + FGG) mutations. The impact of thrombophilia on the risk of VTE recurrence has been examined in several studies. Similar findings were obtained by De Stefano et al., Simioni et al. and Lindmarker et al. [36,37,38] who demonstrated that the presence of both FVL and/or the PTM mutations were associated with increased risk of recurrent DVT. In addition, Prandoni et al. [2] reported that thrombophilia was associated with an increased risk of recurrence among patients with unprovoked VTE, and patients with multiple abnormalities had higher risk for VTE recurrence compared with patients with single abnormality. This result is not supported by any other prospective studies by Baglin et al., Santa Maria et al. and Lijfering et al. [19,39,40]. These contradictory points of view could be attributed to the differences in the study design, definition of the patients (provoked/unprovoked), documentation of the first event as well as the second event, definition of outcome and type and duration of anticoagulant therapy. Very interestingly, by using the 5-SNP GRS, previously validated for first venous thrombosis, our results suggest that the GRS model could be an effective and useful model in risk stratification of VTE patients, and genetic risk profiling of VTE patients could be used for the prediction of recurrence of VTE. In agreement with our results, van Hylckama Vlieg et al. [18] stated that by using this 5-SNP GRS model, they were able to stratify patients into high and low risk of recurrence. This 5-SNP model is sufficient for the prediction of VTE recurrence in both provoked and unprovoked first events. Close to our findings, Ahmad A et al. [35] demonstrated that the 8-SNP GRS had a better performance in risk prediction of VTE recurrence compared to the previously described 5-SNP GRS, and they revealed this improved performance of the 8-SNP GRS with the inclusion of 3 additional SNPs.

## 5. Conclusions

GRS model could be an effective and useful model in risk stratification of VTE patients, and genetic risk profiling of VTE patients could be used for the prediction of recurrence of VTE.

Limitations of the study: The present study has several limitations that should be acknowledged. First, we did not assess other thrombophilic conditions (e.g., antithrombin, protein C and S deficiency). Second, this study involved a relatively small number of patients. Third, our study period was only five years with a short follow-up period; thus, we may have missed recurrent events that occurred afterwards. Fourth, the study revealed a relatively low (~60%) sensitivity of diagnostic measures (compression sonography) for distal DVT. Ultimately, it is a single center study applied to population with similar ethnic background.

## Figures and Tables

**Table 1:** Demographic data of the two studied groups of patients.

	Total (*n* = 224)	Recurrence (*n* = 58)	Non Recurrence (*n* = 166)	Test of Sig.	*p*
**Gender**					
Male	108 (48.2%)	28 (48.3%)	80 (48.2%)	χ^2^ = 0.0	0.991
Female	116 (51.8%)	30 (51.7%)	86 (51.8%)		
**Age (years)**					
Mean ± SD.	51.7 ± 6	52.1 ± 6.3	51.6 ± 6	t = 0.455	0.650
Median (Min.–Max.)	50 (41–66)	51 (41–66)	50 (42–66)		

χ^2^: Chi square test; t: Student *t*-test.

**Table 2:** Genotyping of the five SNPs among the two studied groups of patients.

	Total (*n* = 224)	Recurrence (*n* = 58)	Non Recurrence (*n* = 166)	χ^2^	*p*
**ABO**					
O	71 (31.7%)	15 (25.9%)	56 (33.7%)	χ^2^ = 24.189 *	<0.001 *
A	67 (29.9%)	15 (25.9%)	52 (31.3%)		
B	60 (26.8%)	11 (19%)	49 (29.5%)		
AB	26 (11.6%)	17 (29.3%)	9 (5.4%)		
Non O	153 (68.3%)	43 (74.1%)	110(66.3%)	χ^2^ = 1.231	0.267
**FVR506 Q**					
RR	163 (72.8%)	35 (60.3%)	128 (77.1%)	7.684 *	^MC^*p* = 0.011 *
RQ	60 (26.8%)	22 (37.9%)	38 (22.9%)		
QQ	1 (0.4%)	1 (1.7%)	0 (0%)		
RQ + QQ	61(27.2%)	23(39.7%)	38 (22.9%)	6.095 *	0.014 *
**FIIG20210 A**					
GG	199 (88.8%)	49 (84.5%)	150 (90.4%)	1.498	0.221
GA	25 (11.2%)	9 (15.5%)	16 (9.6%)		
AA	0(0%)	0(0%)	0(0%)		
GA + AA	25 (11.2%)	9 (15.5%)	16 (9.6%)	1.498	0.221
**FG G**					
CC	134 (59.8%)	30 (51.7%)	104 (62.7%)	5.257	0.072
CT	68 (30.4%)	18 (31%)	50 (30.1%)		
TT	22 (9.8%)	10 (17.2%)	12 (7.2%)		
CT + TT	90(40.2%)	28(48.3%)	62(37.3%)	2.135	0.144
**FX I**					
TT	112 (50%)	26 (44.8%)	86 (51.8%)	8.473 *	0.014 *
CT	91 (40.6%)	21 (36.2%)	70 (42.2%)		
CC	21 (9.4%)	11 (19%)	10 (6%)		
CT + CC	112(50%)	32(55.2%)	80(48.2%)	0.838	0.360

χ^2^: Chi square test; MC: Monte Carlo; * Statistically significant at *p* ≤ 0.05.

**Table 3:** Risk allele frequency of the five SNPs among the two studied groups of patients.

	Total (*n* = 224)	Recurrence (*n* = 58)	Non Recurrence (*n* = 166)	χ^2^	*p*
**ABO**					
Allele					
O	269(60%)	56(48.3%)	213(64.2%)	χ^2^ = 9.116 *	0.010 *
Non O	179 (40%)	60 (51.7%)	119 (35.8%)		
**FVR506Q**					
Allele					
R	386(86.2%)	92(79.3%)	294(88.6%)	6.160 *	0.013 *
Q	62(13.8%)	24(20.7%)	38(11.4%)		
**FIIG20210A**					
Allele					
G	423(94.4%)	107(92.2%)	316(95.2%)	1.410	0.235
A	25(5.6%)	9(7.8%)	16(4.8%)		
**FGG**					
Allele					
C	336(75%)	78(67.2%)	258(77.7%)	5.025 *	0.025 *
T	112(25%)	38(32.8%)	74(22.3%)		
**FXI**					
Allele					
T	315(70.3%)	73(62.9%)	242(72.9%)	4.086 *	0.043 *
C	133(29.7%)	43(37.1%)	90(27.1%)		

χ^2^: Chi square test; * Statistically significant at *p* ≤ 0.05.

**Table 4:** Comparison between the two studied groups according to No of risk Allele (GRS).

	Total (*n* = 224)	Recurrence (*n* = 58)	Non Recurrence (*n* = 166)	Test of Sig.	*p*
No of risk Allele (GRS)					
Low risk (0–2)	160(71.4%)	31(53.4%)	129(77.7%)	χ^2^ = 12.398 *	<0.001 *
High risk (3–8)	64(28.6%)	27(46.6%)	37(22.3%)		
Mean ± SD.	2.3 ± 1.2	3 ± 1.9	2 ± 0.7	U = 3631.0 *	0.003 *
Median (Min.–Max.)	2 (0–8)	2 (1–8)	2 (0–4)		

χ^2^: Chi square test; U: Mann–Whitney test; * Statistically significant at *p* ≤ 0.05.

**Table 5:** The Risk of Recurrent VTE Associated With the five SNPs among the two studied groups of patients.

	SNP	*p*	HR (95% CI)	*p*	HR # (95% CI)
**FV**	rs6025	0.019 *	1.877(1.109–3.178)	0.015 *	1.951(1.141–3.338)
**FII**	rs1799963	0.198	1.595(0.784–3.248)	0.207	1.593(0.773–3.284)
**FGG**	rs2066865	0.128	1.492(0.892–2.498)	0.137	1.482(0.883–2.487)
**ABO**	rs8176719	0.239	1.424(0.791–2.563)	0.232	1.431(0.795–2.577)
**FXI**	rs2036914	0.432	1.230(0.733–2.064)	0.416	1.241(0.738–2.085)
**GRS (≥3)**		0.001 *	2.472(1.475–4.145)	0.001 *	2.503(1.486–4.217)

#: Adjusted hazard ratio by age and sex; CI: Confidence interval; LL: Lower limit; UL: Upper Limit; * Statistically significant at *p* ≤ 0.05.

**Table 6:** Kaplan–Meier survival curve for recurrence free survival with GRS.

GRS	Mean	%	Log Rank	
			χ^2^	*p*
Low risk (0–2)	52.163	80.6	12.657 *	<0.001 *
High risk (3–8)	43.078	57.8		

* Statistically significant at *p* ≤ 0.05.

## Data Availability

The data presented in this study are available in References [1,2,3,4,5,6,7,8,9,10,11,12,13,14,15,16,17,18,19,20,21,22,23,24,25,26,27,28,29,30,31,32,33,34,35,36,37,38,39,40]. Below are suggested Data Availability Statements: (1) Data available in a publicly accessible repository. The data presented in this study are openly available in [repository name e.g., FigShare] at [doi], reference number [reference number]. (2) Data available in a publicly accessible repository that does not issue DOIs. Publicly available datasets were analyzed in this study. This data can be found here: [link/accession number]. (3) Data available on request due to restrictions e.g., privacy or ethical. The data presented in this study are available on request from the corresponding author. The data are not publicly available due to [insert reason here]. (4) 3rd Party Data. Restrictions apply to the availability of these data. Data was obtained from [third party] and are available [from the authors/at URL] with the permission of [third party]. (5) Data sharing not applicable. No new data were created or analyzed in this study. Data sharing is not applicable to this article. (6) Data is contained within the article. The data presented in this study are available in this article.
